# Supplementary material for: BcXyl, a β-xylosidase Isolated from Brunfelsia Calycina Flowers with Anthocyanin-β-glycosidase Activity
Source: Int J Mol Sci. 2019 Mar 21;20(6):1423. doi: 10.3390/ijms20061423 (PMC6470699; doi:10.3390/ijms20061423)

**BcXyl, a β-xylosidase isolated from *Brunfelsia Calycina* flowers with anthocyanin-β-glycosidase activity**

**Boyu Dong^1, †^, Honghui Luo^1, 2, †^, Bin Liu^1, 2^, Wenjun Li^1, 2^, Shaojian Ou^2^, Yongyi Wu^2^, Xuelian Zhang^1, 2^, Xuequn Pang^1, 2,^ * and Zhaoqi Zhang^1,^ ***

^1^ State Key Laboratory for Conservation and Utilization of Subtropical Agro-bioresources/ Guangdong Provincial Key Laboratory of Postharvest Science of Fruits and Vegetables/ College of Horticulture, South China Agricultural University, Guangzhou 510642, China;

kuroro1986@163.com (B.D.); hhluo@stu.scau.edu.cn (H.L.)

^2^ College of Life Sciences, South China Agricultural University, Guangzhou 510642, China; bin278083@foxmail.com (B.L.); lookitso@hotmail.com (W.L.), osj1991@163.com (S.O.); zoewu09@foxmail.com (Y.W.); xuelianzhang@scau.edu.cn (X.Z.)

***** Correspondence: zqzhang@scau.edu.cn (Z.Z.); xqpang@scau.edu.cn (X.P.); Tel.: +86-20-8528-2180 (Z.Z.); +86-20-8528-0195 (X.P.)

† These authors contributed equally to this work.


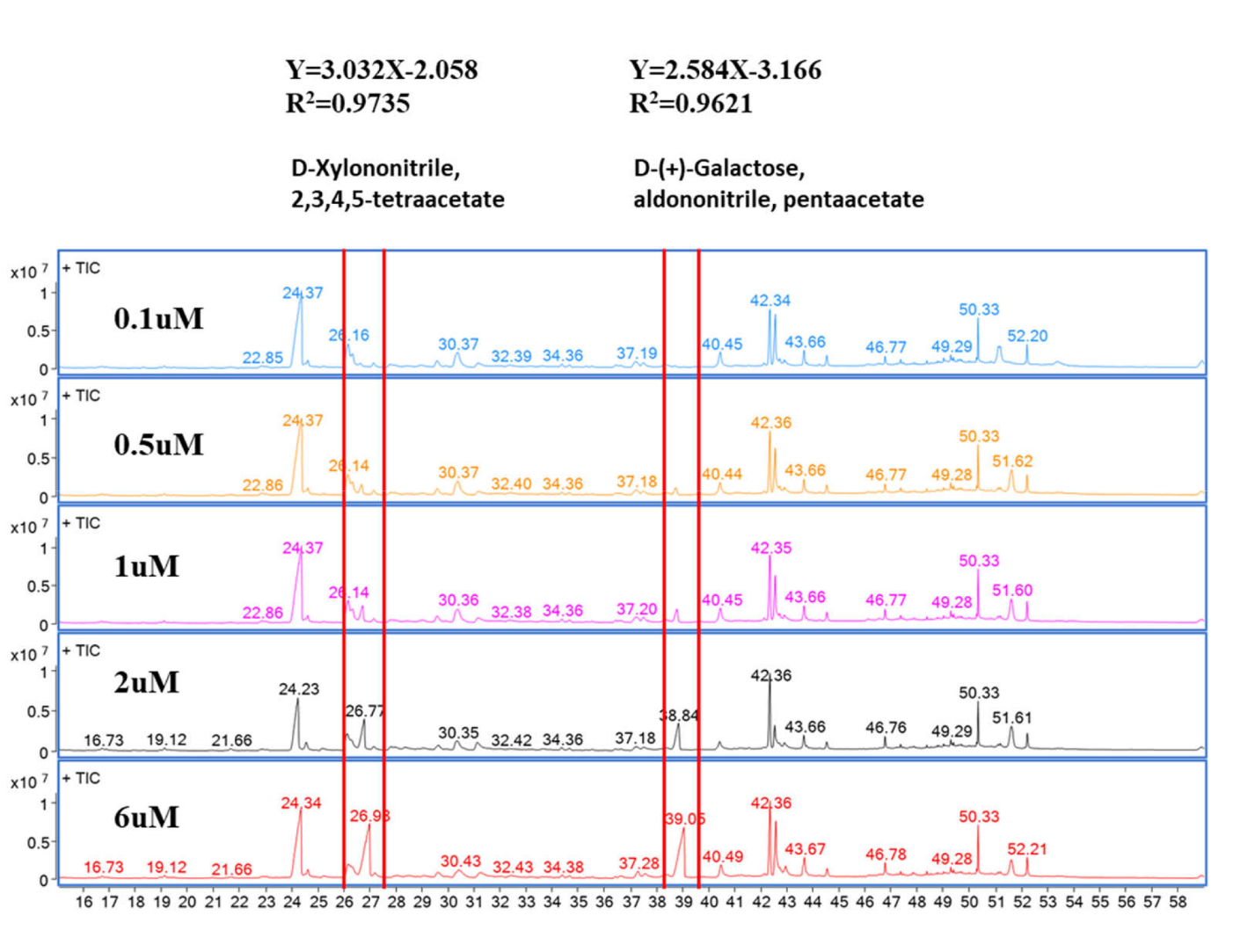


**Figure S1.** **GC-MS detection of the standard monosaccharides and standard curves determination.**


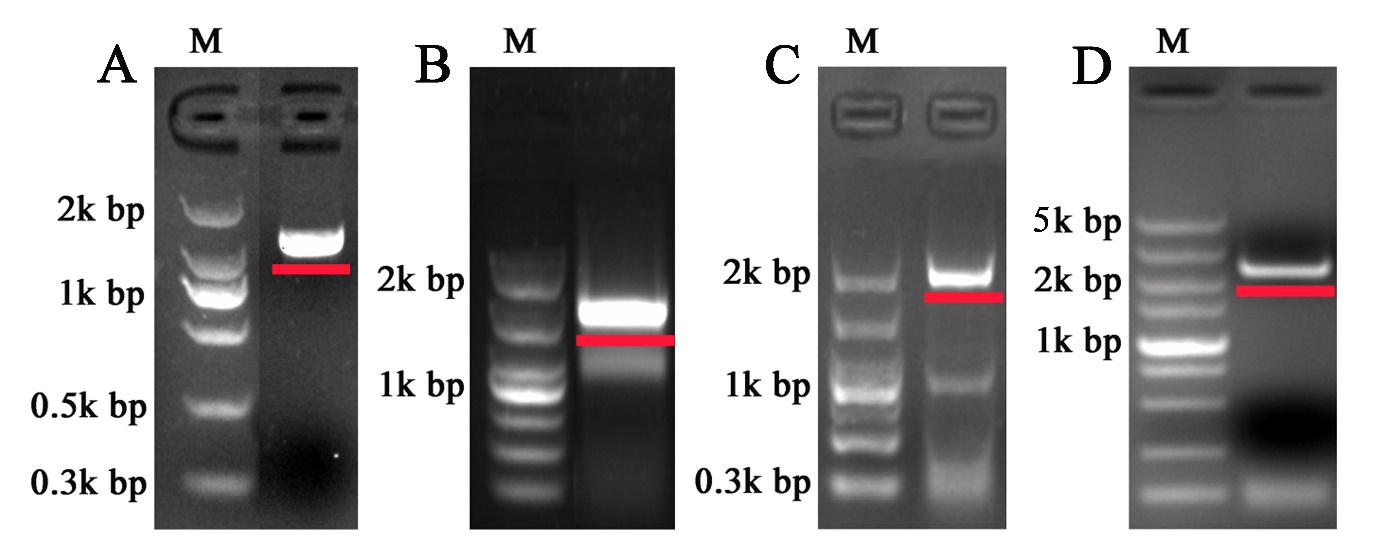


**Figure S2.** **Degenerate primer cloning (A), 5’- RACE (B) and 3’-RACE (C) cloning, Full-length cDNA cloning (D) of the *BcXyl* gene from *Brunfelsia calycina* flowers.** “M” indicates Molecular marker. Red straight line indicates the band containing clone sequences.

**Table S1.** **Primers used for full length cDNA fragment cloning and qRT-PCR analysis of *BcXyl* gene.**


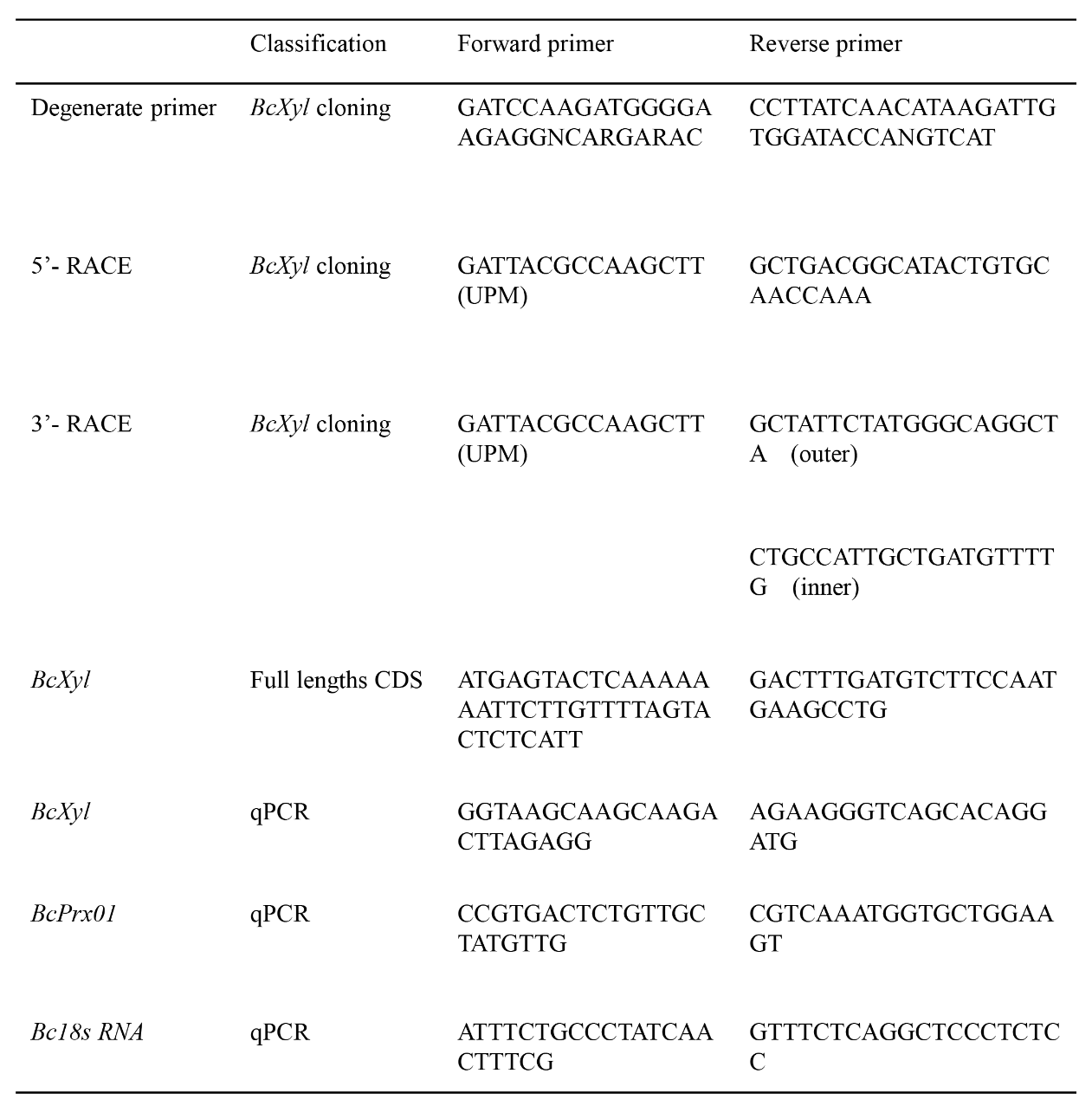

Supplement: Supplementary file 1 [file ijms-20-01423-s001.zip › Supplementary Files/Supplementary-β-xylosidase in Brunfelsia-ijms.docx]
