# Supplementary figures and images for: BcXyl, a β-xylosidase Isolated from Brunfelsia Calycina Flowers with Anthocyanin-β-glycosidase Activity
Source: Int J Mol Sci. 2019 Mar 21;20(6):1423. doi: 10.3390/ijms20061423 (PMC6470699; doi:10.3390/ijms20061423)

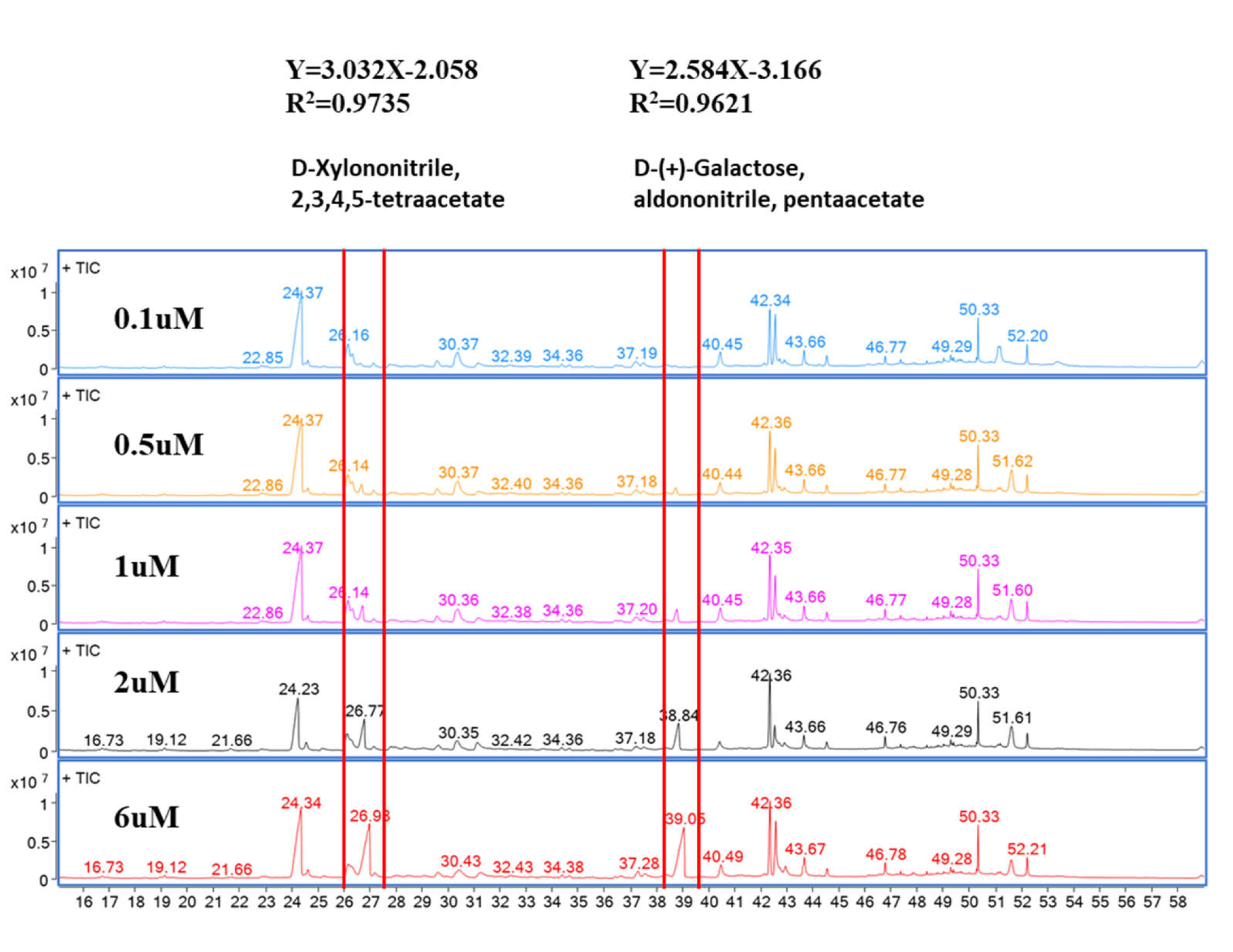

Supplement: Supplementary file 1 [file ijms-20-01423-s001.zip › Supplementary Files/Figure S1.tif]

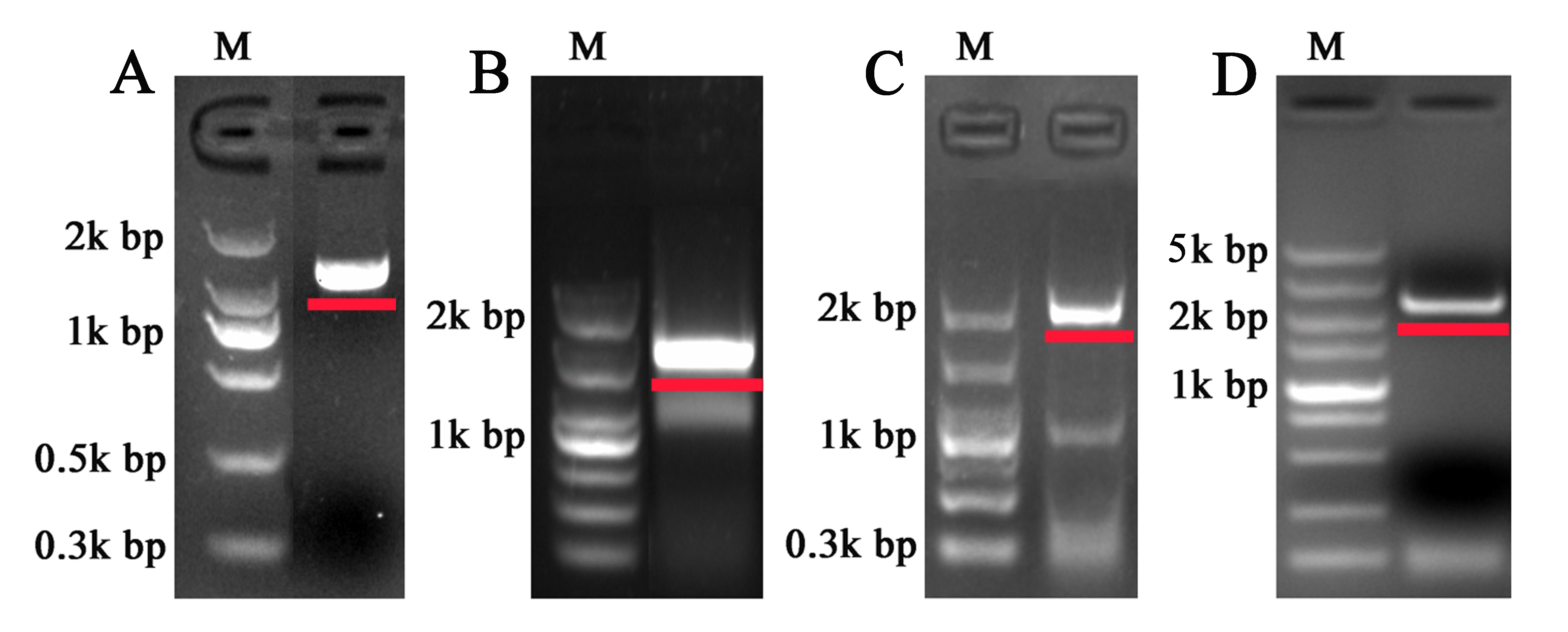

Supplement: Supplementary file 1 [file ijms-20-01423-s001.zip › Supplementary Files/Figure S2.jpg]
